# Determinants for Safety Climate Evaluation of Construction Industry Sites in Saudi Arabia

**DOI:** 10.3390/ijerph17218225

**Published:** 2020-11-06

**Authors:** Anas A. Makki, Ibrahim Mosly

**Affiliations:** 1 Department of Industrial Engineering, Faculty of Engineering—Rabigh Branch, King Abdulaziz University, Jeddah 21589, Saudi Arabia; 2 Department of Civil Engineering, Faculty of Engineering—Rabigh Branch, King Abdulaziz University, Jeddah 21589, Saudi Arabia; ikmosly@kau.edu.sa

**Keywords:** safety climate, evaluation, determinants, construction, Saudi Arabia

## Abstract

The hazardous nature of the construction industry requires giving increasing attention to safety management and the available means to eliminate or reduce the risks of workers’ injuries. Workers in the construction industry of Saudi Arabia face similar daily risks as workers face in other countries. The safety climate significantly influences safety performance, making research in the field of safety climate a vital step toward raising safety levels at construction sites. This study aims at exploring key components of determinants for safety climate evaluation of Saudi Arabian construction sites. Using data collected from 401 industry practitioners, a dimension reduction statistical approach and exploratory factor/principal component analysis were conducted on 13 safety climate factors that were found to significantly correlate with safety climate evaluation of construction sites. The study revealed three key components of determinants for safety climate evaluation of Saudi Arabian construction sites. Notable components are safety commitment, safety interaction, and safety support. Implications of this study include assisting construction industry stakeholders to bolster the safety climate at their construction sites, which should lead to improved safety performance levels.

## 1. Introduction

Occupational safety is a serious global issue in the construction industry [1], mainly due to the industry’s consistently high injury rates. Construction safety is considered to be a complex phenomenon [2], and to effectively engage this complexity, a holistic approach that is more sophisticated than the traditional safety compliance methods is required [1]. Furthermore, the involvement of several stakeholders such as owner, designers, construction managers, and subcontractors adds to this complexity [3]. These stakeholders all have unique views on aspects of safety. Although several important developments have occurred, over the years, in safety management, the construction industry still has high rates of serious and nonserious injuries, as well as fatalities [4]. Whether the construction industry injuries are fatal or not, they continue to remain worryingly high [5,6]. Due to the often hazardous nature of their work, frontline construction workers are constantly exposed to threats and accidents [7]. This scenario is no different in the context of the construction industry in Saudi Arabia. Therefore, the focus should be on the health and safety of these workers to eliminate or at least reduce risk. Thus, there should be a consistent emphasis on safety by construction project management even under intense production pressure [8].

The construction industry is influenced by safety climate in various ways. According to many research studies, maintaining high safety standards is essential at construction sites and the safety climate influences safety performance [5,7,9,10,11,12,13,14]. A site’s safety climate also serves as a safety performance predictor [5,11,15] and encourages employees’ behavior toward safety [16,17,18,19], while improving employees’ precautionary behavior. Furthermore, the safety climate represents an organization’s safety priorities and leads to the exploration of areas of safety enhancement [20], and therefore encourages development and innovation in safety. There is increasing evidence that an improvement in workplace safety is accompanied by improvement in safety practices [21]. Workplace safety requires employers to fulfill health and safety standards at their work location [22], and safety practices are the written guidelines that illustrate how to conduct activities with the least risk influence on people and surroundings [23].

The safety climate and safety behavior at construction jobs are always closely associated [21] and this association is well recognized in the construction industry [24]. This link between safety climate and behavior is important and affects construction site safety. A recent study in Hong Kong resulted in a statistically significant relationship among safety climate, safety behavior, and several personal characteristics [25]. Moreover, a study that took place in China concluded that an organizational safety climate influenced both safety behavior and the safety awareness of the individual workers [19]. To improve safety performance in the construction industry, a more specific approach is needed, where the casual relationship among safety behavior, safety climate, and the personal experience becomes vital to improve safety management strategies [26]. Such an approach would be beneficial to the construction industry in general. A positive safety climate can also potentially reduce risk-taking behavior of construction workers [27], and therefore should result in fewer cases of injuries and accidents. Furthermore, maintaining both a safety climate and safety motivation has been found to positively impact safety behavior [28]. A study in China also confirmed this impact and demonstrated that a coworker’s safety climate assisted the workers to change their risk perception to become positive and as a result motivated them to practise safety behavior [29]. However, the perception of safety climate for construction workers is established at different levels and differs from one group to another [30]. For instance, the perception of safety climate varies among supervisors and workers [31], among workers from different countries, and among workers at the same construction site. Another example was a study that took place in Taiwan which illustrated that, in the construction industry, the management-level staff has a more positive perception of safety climate as compared with the worker-level staff [32]. In addition, variations in safety climate perception levels for workers from different countries have been detected. For instance, a study revealed that the safety climate perception of Chinese workers was higher as compared with Vietnamese workers [33]. Therefore, workers at the same construction site can also have different degrees of safety climate perception, and this has been seen most often on construction projects employing ethnic minority workers. Because it is hard to evaluate ethnic minority perception, this group is often understated in safety climate research [14]. According to statistics, they had a higher injury rate than local workers [14]. This could be due to assigning them to more hazardous tasks or communication barriers. Thus, training and communication are required to influence safety perception. Safety training by recognized safety establishments can raise safety perception among members of an organization [34], because an enhanced safety climate perception can be fulfilled by clear communication of rules [35]. Additionally, effective communication has a significant role in the success of the relationship between safety climate and safety outcomes [36]. For example, a study that took place in the United States of America concluded that better communication among construction workers, which made them more active in providing and receiving safety information, could be achieved through promoting a positive safety climate [37].

Several research studies have investigated safety climate factors in the construction industry from different national and cultural perspectives. A study concluded that cultural adjustment needed to be considered in existing safety climate scales and that these scales could not be generalized over countries and regions [38]. Here, the emphasis was on exploring safety climate factors in the construction industries in different countries worldwide. Generally, there were differences in the safety climate factors identified from one region to another, but common factors did exist among these studies. Furthermore, several researchers have applied factor analysis techniques to explore the relationships among safety climate factors. For example, in the United States, a study that investigated safety climate factors through confirmatory factor analysis, found that the most attention should be given to management commitment and supervisor support as they had considerable influence on other safety dimensions [39]. Another study, which took place in China, found similar results, where the authors used confirmatory factor analysis on safety climate dimensions and concluded that safety management participation and safety personnel backing had the most significant influence on construction teams as compared with other dimensions [40]. A study built on a systematic review of the literature to investigate safety climate factors and the performed factor analysis of various studies concluded minor uniformity on factor importance [9]. However, it was also found that management commitment in the role of the supervisor, as well as workers’ participation and group safety climate were the common topics across the literature [9]. Furthermore, new safety climate factors are still being identified by research studies. For instance, a study identified safety imposition and encouragement as a novel safety climate factor [41], thus, adding to the already known safety climate list of factors. Another study, which was done in the Australian construction industry, concluded that “coworkers’ actual safety response” must be added as a dimension to the group-level safety climate [42]. This emphasizes the significance of continuous research and development in the field of safety climate and its influencing factors, especially in the context of the construction industry in Saudi Arabia.

Therefore, despite the many studies that have explored the components of safety climate in the construction industry, a knowledge gap still exists [40]. Hence, with the motivation of filling this gap and due to the need to develop and enhance the safety climate levels in the Saudi Arabian construction industry, this research explores the key components of determinants for that industry’s safety climate evaluation. Our results contribute to the available research related to safety climate in the Saudi Arabian construction industry and the related geographic region, an lead to an enhanced awareness in this field. This study also expands on the available safety climate knowledge, as the identified components will assist in increasing safety awareness and management. Moreover, construction professionals can benefit from this research outcome and enhance their construction sites by regularly considering and evaluating the identified safety climate determinants. Furthermore, the information provided in this study can be used by governments and private sectors to promote safety in the construction industry.

## 2. Materials and Methods

As mentioned previously, this study is a continuation of the Mosly and Makki study [43]. They conducted an extensive literature review process based on Mosly [44] to extract 18 primary factors generally influencing the safety climate in the Saudi Arabian construction industry. Furthermore, they revealed a specific set of 13 statistically significant correlations between safety climate factors and safety climate evaluation of construction sites in Saudi Arabia (see Table 1).

This study builds on Mosly and Makki [43] using collected data from construction industry practitioners along with the set of factors listed in Table 1. The objective is to explore key components of determinants for safety climate evaluation of Saudi Arabian construction sites. Thus, 13 questionnaire items were designed for the factors listed in Table 1 to measure the employees’ perceptions of the importance of each factor in influencing safety climate at their sites using a 5-point Likert scale (i.e., extremely important, important, neither, unimportant, and extremely unimportant). Sampling was conducted randomly to collect cross-sectional information from 3 large projects in Saudi Arabia using the designed survey questionnaire. Five-hundred site employees were targeted, and 401 employees fully responded (i.e., 80.2% response rate). Sociodemographic information of respondents was also collected. It included information on respondents’ age, nationality, education, occupation, years of experience, and their trade specialty. The sample included construction site employees, ranging in age from 18–20 to more than 50 years old, with the largest group in the sample of employees aged 36–40 years (105, 26.20%). Furthermore, the sample included employees of 9 different nationalities from Sudan, Philippines, Bangladesh, Somalia, Syria, India, Yemen, Egypt, and Pakistan, which represented the largest group of employees in the sample (129, 32.17%). The educational level of employees in the sample ranged from illiterate to bachelor degree holders with almost an even distribution. Additionally, the sample included employees of 6 different occupations, i.e., workers, technicians, supervisors, architects, engineers, and managers, with the largest group in the sample being workers (294, 73.31%). Information on years of experience was also collected, and the sample included groups of employees with experience ranging from 0–5 years to more than 20 years, with the largest group in the sample (134, 33.41%) of employees having 6–10 years of experience. Moreover, the sample included employees working in 14 different trade specialties, i.e., carpenters, blacksmiths, bricklaying, painters, plumbing, cement and concrete, crane operators, surveyors, mechanical, electrical, civil, architects, administration, and safety/quality control. The largest trade specialty group in the sample was carpenters (100, 24.93%). Full ethical approval for the performed survey in this study was granted by the Ethical Committee of the Center of Excellence In Genomic Medicine Research, King Abdulaziz University, (HA-02-J003).

To achieve the objective of this exploratory study, the main components of safety climate evaluation determinants were extracted in a dimension reduction fashion. The statistical approaches, exploratory factor analysis (EFA) along with principal component analysis (PCA), and the varimax orthogonal factor rotation method were used. Additionally, to validate the survey questionnaire items designed for each of the 13 factors presented in Table 1, and to ensure the reliability of the later extracted components, scale reliability using the Cronbach’s alpha (*α*) statistic was analyzed. Data analysis was conducted using the Statistical Package for Social Sciences computer software SPSS version 23.0 (SPSS Inc, Armonk, NY, USA) [45]. The results and discussion of this study are presented next.

## 3. Results and Discussion

Firstly, a reliability analysis was conducted for all items in the survey questionnaire of the 13 factors significantly correlating with safety climate evaluation in construction sites (Table 1). The analysis resulted in a Cronbach’s *α* value of 0.767, which demonstrates a satisfactory level of reliability concerning relevant factors (i.e., >0.70) according to several sources and studies [46,47,48]. Additionally, the reliability analysis did not indicate any increase in the reliability level when any of the tested factors were removed from the survey questionnaire.

Secondly, the results of the performed EFA-PCA with the varimax rotation method [46,49] on the 13 factors listed in Table 1 demonstrated high sampling adequacy indicated by the resulted Kaiser–Meyer–Olkin (KMO) value of 0.810, which was deemed to be acceptable [46,50,51]. Additionally, individual KMO values of each factor were checked and ranged between 0.735 and 0.857, which registered well above the acceptable minimum threshold value of 0.50 [46,52]. Nine items’ KMO values ranged between 0.80 and 0.90, and four items’ KMO values ranged between 0.70 and 0.80. The correlations between factors were also checked using Bartlett’s test of sphericity which resulted in *χ*^2^(78) = 1081.877, *p* <0.001, indicating that they were suitably and sufficiently high for the EFA-PCA. As a preliminary analysis step, individual eigenvalues were calculated to check for the potentially available component of factors within the data (Table 2). Using Kaiser’s criterion of factors extraction (i.e., eigenvalues >1.000) [46], three components were shown to have eigenvalues ranging between 1.037 and 3.486 before rotation (Table 2) and ranging between 1.578 and 2.545 after rotation, as detailed in Table 3. The results also showed that the three extracted components in combination were able to explain a total of 50.647% of the variation, as shown in Table 3.

Furthermore, the conducted correlation analysis between factors resulted in a determinant of correlation matrix that equaled 0.065 > 0.00001, indicating that multicollinearity or singularity in the used dataset was not problematic, according to Field [46]. According to Field [46] and Stevens [53], generally, item loadings that register above the absolute values of 0.40 are considered to be the bare minimum in the clusters. In this exploratory study, only factors with a loading value >0.520 as a cut-off value were retained for interpretation. This is due to the high item loadings ranging between 0.520 and 0.775, as illustrated in Table 3.

To ensure consistency of the extracted factors in terms of their clustering and the resulting number of clusters under two scenarios, i.e., (1) when correlations between components are expected and (2) when components are expected to be uncorrelated, further EFA-PCA analysis was conducted using other factor rotation methods. Orthogonal factor rotation methods such as Equamax and Quartimax, and oblique rotation methods such as direct Oblimin and Promax, were used for the first and second scenarios, respectively. The results of the analysis indicated a high level of consistency and produced identical factor clustering and number of clusters. Therefore, the resulted factor clustering using the varimax rotation method, and its extracted components are retained and considered to be reliable. The results of the varimax rotation method are presented here due to its distinctive ability in maximizing the within-factor loadings’ dispersion [46].

Therefore, based on the fairly large sample size (*n* = 401), and the components satisfying Kaiser’s criterion of extraction based on their calculated eigenvalues (Table 2), three components were retained in the final analysis of this study. Reliability analysis was performed on the extracted components using Cronbach’s α test statistics. The results of the three extracted components, presented in Table 3, show high values of Cronbach’s α of 0.808, 0.817, and 0.773, respectively. All values exceed the threshold of α > 0.70, indicating high levels of reliability [47].

Finally, the three extracted components are considered to be determinants for safety climate evaluation of Saudi Arabian construction sites. Table 3 shows the factor loadings after the varimax factor rotation was performed. The factors that cluster, based on their loadings, in the same components propose the following: Component 1, (named “safety commitment”) signifies determinants related to commitment, risk appraisal, justice, and compliance; Component 2 (“safety interaction”) signifies determinants related to involvement, influence, communication, attitude, and supportive environment; and Component 3 (“safety support”) signifies determinants related to the means of support given to safety by the construction organization. All clustered determinants were directly related to the identified components.

The following five determinants were clustered in the first component of safety commitment: workers’ safety commitment, appraisal of risks and hazards, management commitment to safety, management safety justice, and competence. This component includes determinants and relates to safety commitment, whether this commitment is made by the organization management or the workers. It also includes appraisal of risks and hazards, as well as management safety justice, which is also directly related to a commitment to safety. According to a comparison of safety climate factor structure in the construction industry by Newaz et al. [9], the safety climate factor competence was equivalent to the management commitment. This supports the clustering of competence determinants in the safety commitment component.

The following five determinants were clustered in the second component of safety interaction: workers’ involvement, workmate influences, communication, workers’ attitude toward health and safety, and supportive environment. It can be observed that the clustered determinants in this component are all directly related to interaction concerning safety, whether the interaction is in the form of workers’ involvement, influence, or communication with a coworker or management on safety aspects. Moreover, the workers’ attitudes toward health and safety represent a form of safety interaction. Furthermore, the determinant supportive environment has a similar safety climate factor structure as a workmate’s influence, according to [25].

The following three determinants were clustered in the third component of safety support: education and training; social security and health insurance; and supervision, guidance, and inspection. All of these three determinants are resources of safety support for workers, whether that support is in the form of training, health insurance, or supervision by management.

In comparison with components extracted in other studies, the aforementioned resulted clustering of determinants and the formed components in this study seem to be specific to the Saudi Arabian construction industry context, whereas some of the revealed components in this study are similar to previously reported components in other contexts. For instance, a study that investigated the components of safety climate determinants for construction projects in Pakistan resulted in extracting four components [38]. These components were the following: (1) management commitment and employees’ involvement in health and safety, (2) safety enforcement and promotion, (3) applicability of safety rules and safe work practices, and (4) safety consciousness and responsibility [38]. It can be observed that the component safety commitment was a common component that was identified in this study and the previous study in the context of Pakistan. Another example was a study, which took place in China, that identified six safety climate components [40]. These components were the following: (1) workers’ self-perception of safety, (2) workers’ involvement in safety, (3) co-workers’ interaction, (4) safety environment, (5) safety management involvement, and (6) safety personnel support [40]. Here, more similarities between the extracted components in this study and the previous study in China can be found. The component safety interaction in this study is similar to the reported co-worker’s interaction component. Additionally, the component safety support in this study is similar to the reported component of safety personnel support. Thus, it can be observed that some safety climate dimensions are country/region specific.

Some recommendations can be derived from the revealed three components in this research study. Firstly, to raise safety climate perception in both public and private organizations of the construction industry in Saudi Arabia, it is essential to consider and emphasize the implantation and enhancement efforts of safety climate in the directions of the three identified dimensions. Secondly, safety commitment should be encouraged at all levels of any organization in the construction industry, as it will reflect higher levels of responsibility and obligation in following the available safety standards and guidelines. Thirdly, ensuring sufficient safety interaction will lead to successful collaboration and trust among employees, which is likely to increase productivity. Finally, providing safety support will assist construction site employees in becoming more proactive and confident in their job activities.

## 4. Conclusions

This study aimed at exploring key components of determinants for safety climate evaluation of Saudi Arabian construction sites. Exploratory factor analysis was conducted on survey questionnaire items based on 13 safety climate evaluation factors. Three main components were extracted using PCA and varimax with Kaiser normalization factor rotation method. The analysis results clustered all determinants for safety climate evaluation of construction sites into three key components. These components of determinants are safety commitment, safety interaction, and safety support. The study concludes that these three key components are significant to the Saudi construction industry. The set of safety climate evaluation determinants revealed in this study can help construction industry stakeholders to enhance the safety climate on construction sites, which can translate to improved safety performance levels. The components of determinants found in this study are derived from patterns within the dataset used. Thus, one future research direction should be to conduct the study in other spatial and temporal contexts. Furthermore, the revealed structure of components of determinants can guide the development of a safety climate evaluation prediction model for another future research direction.

## Figures and Tables

**Table 1:** Significant factors for safety climate evaluation (source Mosly and Makki [43]).

No.	Factor
F01	Workers’ safety commitment
F02	Appraisal of risks and hazards
F03	Management commitment to safety
F04	Management safety justice
F05	Competence
F06	Workers’ involvement
F07	Workmate influences
F08	Communication
F09	Workers’ attitude toward health and safety
F10	Supportive environment
F11	Education and Training
F12	Social security and health insurance
F13	Supervision, guidance, and inspection

**Table 2:** Eigenvalues and the extracted number of components before factors rotation.

Component Number	Eigenvalue
1	*3.486*
2	*2.061*
3	*1.037*
4	0.917
5	0.843
6	0.755
7	0.740
8	0.675
9	0.566
10	0.520
11	0.502
12	0.467
13	0.431

Italicized font represents extracted components satisfying Kaiser’s criterion (eigenvalues >1.000).

**Table 3:** Summary of exploratory factor analysis results for the determinants of safety climate evaluation (*n* = 401).

No.	Factor	Rotated Factor Loadings		
		Component 1	Component 2	Component 3
F01	Workers’ safety commitment	*0.721*	−0.028	0.188
F02	Appraisal of risks and hazards	*0.677*	0.153	0.076
F03	Management commitment to safety	*0.627*	0.050	0.268
F04	Management safety justice	*0.606*	0.001	0.325
F05	Competence	*0.600*	0.233	−0.159
F06	Workers’ involvement	0.067	*0.775*	−0.095
F07	Workmate influences	−0.130	*0.747*	0.246
F08	Communication	−0.017	*0.680*	0.249
F09	Workers’ attitude toward health and safety	0.275	*0.640*	−0.070
F10	Supportive environment	0.195	*0.520*	0.075
F11	Education and training	0.322	−0.033	*0.698*
F12	Social security and health insurance	−0.005	0.269	*0.618*
F13	Supervision, guidance, and inspection	0.454	0.066	*0.567*
Eigenvalues		2.545	2.462	1.578
% of Variance		19.576	18.936	12.135
*α*		0.808	0.817	0.773

Extraction method, PCA and rotation method, varimax with Kaiser normalization. Rotation converged in six iterations. Note: Factor loadings greater than or equal to an absolute value of 0.52 appear in italicized font.
